# Maillard reaction products and potatoes: have the benefits been clearly assessed?

**DOI:** 10.1002/fsn3.283

**Published:** 2015-09-17

**Authors:** DeAnn J. Liska, Chad M. Cook, Ding Ding Wang, John Szpylka

**Affiliations:** ^1^ Biofortis Research211 E. Lake St.AddisonIllinois 60101; ^2^ D&V Systematic Consulting1945 Eastchester Rd.Apt 26DBronxNew York 10461; ^3^ Silliker, a Mérieux NutriSciences CompanyChicagoIllinois 60601

**Keywords:** Advanced glycation end products, browning reaction, fiber, potassium, *Solanum tuberosum*

## Abstract

Cooking foods affords numerous food safety benefits. During heating, Maillard reaction products (MRPs) are formed. MRPs contribute sensory aspects to food, including color, taste, and texture. One MRP, acrylamide, has been implicated in negative health outcomes; however, emerging data suggests MRPs may also deliver certain health benefits. The food industry has taken steps to decrease acrylamide formation, but the perception that high levels of acrylamide compromise the nutritional benefit of certain foods has continued. Potatoes are susceptible to MRP formation during cooking but also are considered an affordable, high nutrient content food. In particular, potatoes contribute significantly to fiber and potassium intakes in the U.S. population, two nutrients of need. How, then, should potatoes be judged for effects on health? A structured evidence assessment was conducted to identify literature, specifically clinical trials, on MRPs from potatoes and health, as well as nutritional contribution of potatoes. The results indicate limited human clinical data are available on negative health outcomes of potato‐based MRPs, whereas potatoes are important contributors of key nutrients, such as fiber and potassium. Therefore, a balanced benefit‐risk approach is warranted in order to assure that decreasing consumption of certain foods, like potatoes, does not lead to unintended consequences of nutrition inadequacies.

## Introduction

Cooking food is considered to be the most effective food safety measure to reduce contaminants. The Maillard reaction, or nonenzymatic browning [reviewed in detail elsewhere (Birlouez‐Aragon et al. 2010a; Van Lancker et al. 2011; Parker 2013)], occurs in foods during heating, and results in literally hundreds of different intermediates and end products, called Maillard reaction products (MRPs). Some MRPs have been shown to have in vitro antioxidant activity and/or deter oxidation of unsaturated fatty acids in foods. It is estimated that a person eating a Western diet may consume from ~1 to 10 g or higher of MRPs per day, mainly in the forms of N‐*ε*‐(6)‐Carboxymethyllysine (CML) and pyrraline, although the actual intakes are not known (Ames 2007; Fogliano and Morales 2011).

Many MRPs are large polymers and it is estimated that up to 80% escape digestion. Emerging science suggests these large molecules may be fermented by bacteria in the colon, leading to potentially bioactive metabolites. In particular, the class of MRPs known as melanoidins, high molecular weight, brown‐colored MRP end products found in roasted coffee or bread crusts, has obtained the most interest (Delgado‐Andrade 2014). Overall, the data support that the polysaccharide‐rich melanoidins found in coffee and cereal grains may be preferentially metabolized by bifidobacteria, whereas protein‐rich melanoidins may be substrates for potentially harmful protein metabolizing bacteria that are predominantly present in the descending tract of the colon (Tuohy et al. 2006; Mills et al. 2008; Summa et al. 2008; Moreira et al. 2012). Evidence with melanoidins also suggests they may have chemopreventive activities, antioxidant activities, and anti‐hypertensive actions (Chen and Kitts 2011a,b; Zeng et al. 2011; Moreira et al. 2012; Vitaglione et al. 2012; Delgado‐Andrade 2014; Kanzler et al. 2014).

Some of the small molecule MRPs, such as CML, acrylamide, and pyrraline are generally thought to be bioavailable, although the majority appear to be rapidly excreted (Ames 2007). One MRP, acrylamide, has been implicated in negative health effects and has gained much attention (Pruser and Flynn 2011) but less information is available on health impacts of other MRPs in humans. Additionally, although in vitro and animal studies on MRPs suggest potentially beneficial effects on various health outcomes, the effects may not translate to humans. Therefore, assessment of data in humans is necessary to understand the actual impact of MRPs on health outcomes.

Potatoes, a food susceptible to MRP formation during cooking, have been a staple food in populations across the world for over 10,000 years (Camire et al. 2009). Potatoes are an affordable food option and are unique amongst vegetables in that they are a carbohydrate‐rich food, but also provide beneficial fats (MUFA and PUFA), and are particularly noted for their contribution of key nutrients such as fiber and potassium (Freedman and Keast 2011; Weaver and Marr 2013). Despite the potential benefits associated with consumption of nutrients found in potatoes, the role of potatoes in a healthy diet is controversial (King and Slavin 2013; Ouhtit et al. 2014). In particular, diets such as the “meat and potatoes” diet, and diets that include specific processed forms of potato products such as chips or French fries, are called out as representing unhealthy dietary patterns (King and Slavin 2013; Ouhtit et al. 2014). It remains unclear from discussions to date, however, whether the issue relates specifically to potatoes. Thus, this review was conducted using an evidence mapping approach to understand the balance of science available for assessing benefit versus risk specifically related to MRPs and potatoes. To our knowledge, this is the first attempt to characterize the breadth and scope of human clinical research on this topic.

## Methods

We employed evidence mapping techniques using a bottom‐up approach, in which we started with a broad question and search strategy to identify all published literature. Study designs, populations, interventions, and outcomes were collected to provide a better understanding of the existing studies. The literature was then categorized and characterized.

The structured literature reviews on human health outcomes related to MRPs and/or potatoes were conducted essentially as outlined in the Institute of Medicine's Standards for Systematic Reviews (Moher et al. 2009). Literature searches were conducted in Medline^®^ (January 1994 to April 2014) and Scopus^®^ databases. Studies published in English language were screened to identify articles relevant to the inclusion/exclusion criteria. Reference lists of selected reviews were also screened for additional publications. No gray literature or unpublished data were included.

The assessment of MRPs and human health outcomes was conducted as a systematic literature search. The detailed methodology and the evidence map tables can be found in the Appendix S1. Search terms included “MRPs”, “maillard reaction”, “advanced glycation end products”, “CML”, “melanoidins”, “pentosidine”, and “pyrraline”, as well as terms representing health outcomes. In addition, studies were included if they had a randomized controlled trial (RCT), cohort, case report, or cross‐sectional design, and included an assessment of MRPs or a food with MRPs. Only studies with both an MRP‐related term and a health outcome were selected for abstract review. Studies specifically on acrylamide were included in the search and initial review, but generally excluded from this report due to the availability of numerous recent reviews. The exception was if the study was a clinical trial and/or included assessment of other MRPs with acrylamide.

The potato evidence assessment included a general review of the literature on potatoes and nutrition as well as a systematic review of literature specific to potato MRPs and human health. The general review on potatoes and nutrition was conducted using the search terms “potato(es)” and “white vegetables” singly and in combination with “nutrient intakes”. Studies were included if they were conducted in humans and contained an assessment of dietary intake of potatoes. Reviews and primary literature were included. A systematic search (Appendix S1) was also performed to identify human studies on MRPs from potato(es). For this search, methods followed those for the systematic review of MRPs and health outcomes with the additional search terms of “potato(es)” and “*Solanum tuberosum*”. In addition, only studies that reported a dietary assessment were included.

## Results

### MRPs and health outcomes

The literature search for MRPs and health outcomes generated numerous studies on analytical systems from foods and in vitro or food safety and quality effects of MRPs. With respect to studies on the putative benefits and risks to human health from MRPs in foods, the literature was more limited. Eighteen publications (Vlassara et al. 2002; Uribarri et al. 2003, 2011; Vesper et al. 2005; Seiquer et al. 2006; Negrean et al. 2007; Abramsson‐Zetterberg et al. 2008; Stirban et al. 2008, 2013; Garcia et al. 2009; Mesias et al. 2009; Naruszewicz et al. 2009; Birlouez‐Aragon et al. 2010b; Delgado‐Andrade et al. 2011, 2012; Harcourt et al. 2011; Vikstrom et al. 2011; Correa et al. 2012) on clinical trials that included a health outcome and dietary MRP were identified, with six of these considered duplicate publications (Stirban et al. 2008; Garcia et al. 2009; Mesias et al. 2009; Delgado‐Andrade et al. 2011, 2012; Vikstrom et al. 2011) (Table**** 1). The majority of the clinical trials were conducted on mixed diets that altered the cooking conditions, with 4 reporting potatoes in both the test diet and control diet (Seiquer et al. 2006; Negrean et al. 2007; Abramsson‐Zetterberg et al. 2008; Naruszewicz et al. 2009). Two studies examined the effects of beverages containing differing amounts of MRPs on health outcomes; one was conducted on differing roasts of coffee (Correa et al. 2012) and one was on a purified protein (beta‐lactalbumin) (Stirban et al. 2013).

**Table 1:** Clinical trials on MRPs and potential health outcomes published from January 1994 to April 2014

Citation	Study design	Sample size, population, country	%F	Age (y)	TestFood/Diet	ControlFood/Diet	Duration	Findings
Mixed Diets Containing Potatoes								
Seiquer, et al.*AJCN*. 2006;83:1082–1088	RCTX‐overControlled diet	• *N* = 18• Healthy children• Spain	0%	12.4 ± 0.34	Brown diet (BD) MRP enriched (fried & breaded foods, corn flakes, chocolates, baked products)	White diet (WD) Similar foods and energy/nutrient contents as brown diet. Free as much as possible of MRPs	2 week/diet40–days washout	Nutrient bioavailability study. Significantly lower absorption and higher fecal excretion of nitrogen, although modest effect. Suggests some impact on protein availability from heat treatment. No significant effects on plasma protein profile, however, suggesting no effect on protein metabolism. Comments: Potatoes on both diets (boiled/baked vs. fried) and differences in the food forms. Although diets matched as much as possible, some differences in nutrient composition (e.g., protein lower (not significant) and fat higher (*P* < 0.05) for BD compared to WD)
**Four studies were a re‐analysis of data from Seiquer,** 2006 **(above) and each cross‐referenced Seiqer,** 2006 **as the primary study (see above for caveats)** Garcia, et al. *Mol Nutr Food Res*. 2009;53:1551–60 ○ Findings: Iron intakes similar between BD and WD, but significantly lower apparent iron absorption and retention and higher excretion on BD compared to WD. Suggests MRPs can affect iron bioavailability. Delgado‐Andrade, et al. *Nutrition*. 2011;27:86–91 ○ Findings: Phosphate intakes similar between BD and WD and were higher than dietary recommendations. Non significant increase in fecal phosphate excretion, with significant decrease in apparent absorption, however, no differences in phosphate‐related serum parameters between diets. Mesias, et al. *J Agric Food Chem*. 2009;57:9532–38. ○ Findings: Calcium intakes essentially the same for BD and WD. No significant differences in calcium utilization or biochemical indices of bone metabolism except deoxypyridinoline, which may be related to adolescent bone development (e.g., inconclusive). Delgado‐Andrade, et al. *Amino Acids*. 2012;43:595–602. ○ Findings: CML intake ~2× higher, and fecal excretion ~3× more with BD than WD. Considering fecal and urinary excretion together, both WD and BD resulted in similar proportional excretion, at 45.1% and 49.7%, respectively. Results suggest ~50% of ingested CML may be metabolized (e.g., microbiota) and/or absorbed.								
Negrean, et al.*AJCN*. 2007;85:1236–1243	RCTSingle‐blindX‐over	• *N* = 20• Hospital inpatients with T2DM• Germany	30%	55.4 ± 2.2	High MRP meal (580 kcal)• Food was fried or broiled:○ 200 g chicken○ 150 g potatoes○ 100 g carrots○ 200 g tomatoes○ 15 g vegetable oil	Low MRP meal (580 kcal):• Same foods as high MRP prepared by steaming or boiling	6‐days standardized diabetes diet.Test diets on day 4 and 6	Flow‐mediated dilation (FMD) decreased on both diets, although significantly more after high MRP diet (*P* < 0.01). No significant differences in glucose or insulin. Comments: Heating can affect other factors (vitamins, antioxidants) that have been shown to influence FMD. Potatoes included in both diets.
**One study was a possible duplicate publication with Negrean,** 2007 **(above) that used the same study design and diet intervention, and which cross‐referenced Negrean,** 2007. **However, the study population and demographic/disease characteristics were nearly identical, with apparent differences in one subject per study (e.g., Negrean reported 14 men and 6 women; whereas Stirban reported 13 men and 7 women).** Stirban, et al. *Ann NY Acad Sci*. 2008;1126:276–79. ○ Findings: increased excretion of urinary CML, while serum adiponectin and leptin significantly decreased, and vascular cell adhesion molecule 1 increased (*P* < 0.05).								
Vesper et al. Adv Exp Med Biol. 2005;561:89–96	CTSingle arm	• *N* = 6• Healthy, nonsmokers• USA	NR	≥18	3 oz potato chips/d (21 oz total)	None	7 days	MRP bioavailability study. Exposure to acrylamide was 1.9 µg/kg/day, which is higher than FDA estimated average of 0.4 µg/kg/day. Acrylamide and glycindamide adducts could be detected in serum, but none reached the level expected from high exposure to tobacco smoke. High intra and inter‐subject variability compared to signal. Comments: Substudy of a larger Center for Disease Control (CDC) study that only assessed biomarkers; no health outcomes. Potato chip intake ~3× higher than average consumption, and acrylamide content of potato chips also ~3× higher than average. Therefore, exposure does not reflect average exposure.
Abramsson‐Zetterberg, et al.*Mutation Res*. 2008;653:50–56	RCTParallel	• *N* = 24• Healthy• Sweden	58%	24–60	High‐heated food diet (HHF)Subjects counseled to choose fried foods; supplied with French fries, potato crisps, biscuits, and crisp bread.	Low‐heated food diet (LHF) Subjects counseled to minimize heated foods; supplied with fresh potatoes, buns, and white bread.	4 d	Significant increase in frequency of micronucleated transferring‐positive reticulocytes after HHF diet. Differences in macronutrient content of the diets, particularly fat, which was higher in the HHF diet.
Naruszewicz, et al. AJCN. 2009; 89:733–777	CTSingle arm	• *N* = 14• Healthy• Sweden or Poland	57%	22–56	160 g Potato Chips (PC)878 kcal1374 mg sodium157 mcg acrylamide	400 g Boiled Potatoes (BP)With fat and salt in amounts found in potato chips diet; subjects instructed to avoid potato chips	2 week run‐in on boiled potatoes; 28‐days on PC, then 28‐days on BP	Increased oxidized LDL, hs IL‐6, hsCRP, and γ‐glutamyltransferase with PC diet. Transient effect with no significant changes at the end of study. Smokers (*n* = 6) assessed separately from nonsmokers (*n* = 8) and showed larger responses. Comments; Not randomized.
**As stated by study authors, one publication was a re‐analysis of a combined subset of data from two clinical trials Abramsson‐Zetterberg,** 2008 **(Study 1) and Naruszewicz (Study 2) and was considered a duplicate publication in the evidence map** Vikström, et al. *Toxicol Sci*. 2011;119(1):41–49. ○ Findings: This study compared AUC values of acrylamide and glycidamide Hb adducts in humans after high (11 lg/kg bw) or medium (2.5 lg/kg) acrylamide intake from food with similar values from rodent carcinogenicity tests. This biomarker study suggests Hb adducts of acrylamide and glycidamide in humans may be a useful marker of exposure to dietary AA.								
Mixed Diets – Potatoes Not Included or Not Reported/Unknown								
Birlouez‐Aragon, et al. *AJCN*. 2010b;91:1220–1226	RCTX‐over	• *N* = 62• Healthy • France	50%	18–24	Standard diet (STD)rich in MRP (coffee, grilled/roasted foods, corn flakes, dry cookies, well‐baked bread)	Steamed diet (STMD) low in MRP (raw foods, team, mildly baked bread, sponge cake, steamed corn flakes)	1 week/diet10‐days washout	Significantly higher total and HDL cholesterol on STD, no difference in LDL cholesterol. HOMA score significantly higher on STD. Comment: Significant differences in total kcal, carbohydrates, fats, and vitamin C on the different diets. Change in body weights not provided.
Harcourt, et al.*Kidney Intl*. 2011;80:190–198	RCTX‐over	• *N* = 11• Generally healthy overweight & obese• NR	0%	30 ± 9	High MRP meal • Food prepared by frying, toasting, heating	Low MRP meal• Matched in kcal and foods to high MRP meal; preparations changed to steaming, poaching, fresh/raw	2 week/diet4 week washout	No differences in total cholesterol or fasting glucose. Significantly higher urine 8‐isoprostane and plasma monocyte chemotactic protein‐1, and lower macrophage inhibitory factor on high MRP. Comments: Relatively short‐term intakes.
Uribarri, et al.*J Am Soc Nephrol*. 2003;14:728–731	RCTParallel	• *N* = 18• Stable renal failure patients; nondiabetic • USA	67%	NR	High MRP dietPatients instructed to roast, broil, and oven fry foods	Low MRP dietPatients instructed to boil, poach, steam, stew foods and avoid fried foods	4 week	MRP bioavailability study. Higher serum CML associated with the high MRP diet period, as well as serum AGE levels, suggesting dietary MRPs can contribute to serum AGE in renal failure patients. Comments: Several differences in diet changes from baseline between the two interventions, including total energy, fat, carbohydrate, and protein.
Vlassara, et al.*Proc NatlAcad Sci USA*. 2002; 99:15596–15601.	Study 1 RCTX‐overStudy 2RCTParallelControlled diet	Study 1:• *N* = 11Study 2: • *N* = 13Both studies:• Nonsmoking T1DM & T2DM adults• USA	NR	1: 52 ± 22: ~62	High MRP dietFoods prepared with high cooking times; followed NCEP Step 1 & AHA plans for weight maintenance; MRP content 16.3 ± 3.7×10^6 ^U/day	Low MRP dietComparable foods to high MRP diet with lower cooking times; MRP content 3.7 ± 1.2 × 10^6^ U/day	Study 1:2 week/diet1–2 week washoutStudy 2: 6 week	Significantly higher serum CML during high MRP diet. Changes in blood inflammatory markers suggested pro‐inflammatory response during high MRP diet. No differences in lipids, although significantly more AGE‐modified LDL components during high MRP diet. Comments Meals appeared to vary in food type, and thus likely varied in fat, protein, etc. Study did control for energy intake per subject for weight maintenance.
Uribarri, et al. *Diabetes Care*. 2011; 34:1610–1616.	RCT	• *N* = 36• T2DM (16) and health (16); age‐matched• USA	78%	>60	High MRP diet *n* = 6 T2DM; Subjects' usual diet rich in MRP (dietary MRP intake≥20 AGE Eq/day)	MRP‐restricted diet *n* = 12 T2DM and 16 healthy; isocaloric to high MRP diet; boil, poach, stew, or steam food; limited MRP intake by ~40–50% of usual diet	4 month	No significant changes in healthy controls for any markers except lower serum CML and urinary 8‐isoprostane with the MRP‐restricted diet.Differences were identified with diabetic subjects, including decreased HOMA scores, leptin levels, and plasma insulin, however, adiponectin was ~2× higher with MRP‐restricted diet. Comments: Diets not well described.
Beverages (not Potato‐Based)								
Corrêa et al.*Plant Foods Hum Nutr*. 2012;67:277–282	RCTX‐over	• *N* = 20 Healthy, usual coffee drinkers• Age 49 ± 9• Brazil	70%	49 ± 9	Higher MRP medium roast coffee (~482 mL/day)	Lower MRP medium light roast coffee (~482 mL/day)	1 week run‐in 4 week/dietNo washout	Both coffees resulted in significant increases in plasma total antioxidant capacity, ORAC, and several other antioxidant markers.The lower MRP roast delivered more chlorogenic acid and less caffeine than the higher MRP roast.
Stirban, et al.*Diabetes Care*. 2013;36:1278–1282.	RCTDouble‐blindX‐over	• *N* = 19• T2DM patients• Germany	NR	36–66	MRP beverage• Beta‐lactoglobulin and dextrose heated beverage• 120,000 U CML/serve	Control beverage• Prepared the same as test beverage but without dextrose• 19,400 U CML/serve	One day – single dose7‐days washout	Significantly lower flow‐mediated dilation and plasma CML concentration at 90 min post consumption but not at 180 min for MRP beverage. Comments: Single dose in patients with frank diabetes.

AHA, American Heart Association; AJCN, *Am J Clin Nutr*; BD, brown diet; Ca, calcium; CML, N‐*ε*‐carboxymethyl‐lysine; CT; controlled trial; d, day; Eq, equivalents; g, gram; HHF, high‐heated food; HOMA, homeostasis model assessment; kcal, kilocalorie; LDL, low‐density lipoprotein; LHF, low‐heated food; mcg, microgram; mg, milligram; mo, month; MRP, Maillard reaction product; NCEP, National Cholesterol Education Program; NR, not reported; ORAC, Oxygen Radical Absorbance Capacity; RCT, randomized clinical trial; STD, standard diet; STMD, steamed diet; T1DM, T2DM, type 1 or 2 diabetes mellitus; U, units; wk, week(s); WD, white diet; X‐over, crossover; y, years.

Many of the clinical trials referred to diets as containing advanced glycation end‐products (AGEs). For consistency in terminology, AGEs was changed to MRPs in our evidence map.

The primary focus of research on health impacts of food‐based MRPs related to acrylamide and cancer risk. Acrylamide has been classified as a ‘probable carcinogen,’ and when administered at very high levels is a causative agent for nervous system damage. Acrylamide was first reported as present in some processed foods in 2002, which led to a worldwide effort to decrease its formation during cooking (Fleck 2002; Granda et al. 2004; Pruser and Flynn 2011; Halford et al. 2012; Lineback et al. 2012; Arvanitoyannis and Dionisopoulou 2014; Xu et al. 2014). Three recent reviews have summarized the epidemiological evidence on association of acrylamide with risk of certain cancers (Hogervorst et al. 2010; Pelucchi et al. 2011; Tessier and Birlouez‐Aragon 2012). In addition, Lipworth et al. (2012) published a qualitative review using more restrictive criteria. Our evidence mapping found the same cohorts and cross‐sectional studies (with the exclusion of infant studies), as included in these reviews. In addition, we identified the U.K. Women's Cohort study (Burley et al. 2010), the Baltimore Longitudinal Study of Aging (Semba et al. 2009), and a case–control study in Taiwanese subjects (Chao et al. 2010) (Appendix S1). We did not include some of the earlier publications [from Mucci et al. (case–control studies) and Wilson et al. (Nurses' Health Study II, Sweden Prostate Cancer Cohort)], which are referenced in previous reviews (Lipworth et al. 2012). Furthermore, it should be noted that several expert panel reports on acrylamide [e.g., HEATOX (2007)] are available, but were not specifically identified in our general review of the peer‐reviewed scientific literature. The vast majority of these reports do not include discussions of MRPs beyond acrylamide, and a detailed discussion of acrylamide is beyond the scope of our assessment.

In general, the human studies reporting a relationship between dietary acrylamide and increased risk of cancer are controversial (Hogervorst et al. 2010; Pelucchi et al. 2011). Hogervort et al. (2010) reported that most of the case–control studies did not observe associations between dietary acrylamide intake and increased risk of several cancer types (Hogervorst et al. 2010). However, data from some prospective cohorts suggested dietary acrylamide exposure may be associated with increased risk of postmenopausal endometrial and ovarian cancers, with weaker evidence for renal cell cancer, estrogen (and progesterone) receptor‐positive breast cancer, and oral cavity cancer. Interestingly, these authors also noted acrylamide intake was associated with a reduced risk of many other cancers including lung and bladder cancers in women, and prostate and oro‐ and hypopharynx cancers in men. In contrast, Pelucchi et al. (2011) failed to find an increased risk for most types of cancer from exposure to acrylamide (Pelucchi et al. 2011). The divergent findings of these two review papers, which essentially analyzed the same evidence, suggests the need for more prospective cohort studies that are adequately controlled for confounding and have a sensitive measure to assess dietary acrylamide intakes.

Challenges have been reported in accurately identifying the MRP content of foods, particularly acrylamide, due to the effect that variable food preparation methods has on the type and amount of MRPs formed. The studies discussed above commonly assessed mixed diets with differences in cooking processes (frying and/or baking vs. steaming, boiling, and/or raw foods) (Hogervorst et al. 2010; Pelucchi et al. 2011). Additionally, dietary assessment methods and MRP food databases used may vary considerably and exposure measurement can be imprecise (Dybing et al. 2005; Birlouez‐Aragon et al. 2010a). As noted by Freisling et al. 2013; in an analysis of 24‐h diet recall data, these instruments are not typically designed to gather information related to food processing, such as cooking methods and extent of cooking (Freisling et al. 2013). In addition, it is difficult to quantify the confounding by acrylamide from cigarette smoking and other environmental sources, which contribute higher levels than found in the diet (Pruser and Flynn 2011). Therefore, data on MRP intakes across populations likely do not accurately reflect intakes when dietary self‐report is the primary assessment tool. Furthermore, quantifying dietary acrylamide intakes in large population studies and comparing dietary acrylamide intake estimates from one analysis to another is not reliable, if not unwarranted.

Another complication in assessing the literature on food‐based MRPs and health is the presence of nonenzymatic, chemical modification of in vivo proteins by sugars. The products resulting from this reaction are called advanced glycation end products (AGEs). The first observation of AGEs occurred with the discovery of glycated hemoglobin (HbA_1c_), which is used today as a marker for diabetes (Henle et al. 1996). A relationship has been established between increases in AGEs and presence of age‐related diseases, such as diabetes and cardiovascular disease (Delgado‐Andrade 2014). However, the investigation into whether food‐based AGEs demonstrate similar associations with disease conditions is still in its infancy. Only a handful of studies that clearly identified specific food‐based MRPs have been conducted in human subjects. Thus, the role of food‐based MRPs in health is still an emerging science.

### Potatoes and nutrition

The nutritional contribution of potatoes has been recently thoroughly reviewed, and a summary of reviews on potatoes and nutrient intakes is provided in Table**** 2. In general, potatoes have been reported to provide more nutrients per calorie than most other staple foods (Freedman and Keast 2011). It is also notable that potato products deliver most of their fat in the form of MUFA and PUFA. Fried potatoes contribute only ~2.0% to the average saturated fatty acid intake of the U.S. population, and are not among the top 10 contributors to saturated fatty acid intake (U.S. Department of Agriculture and U.S. Department of Health and Human Services, 2010). In addition, frozen potato products have been shown to contain <0.5 g/serving of *trans* fat (Doell et al. 2012).

**Table 2:** Major reviews and/or meta‐analyses relevant to potatoes and potato nutrition

Citation	Comments
Literature Reviews	
Camire, et al. Potatoes and Human Health. *Crit Rev Food Sci Nutr*. 2009; 49:823–840.	Review of potatoes in the food supply, including potato nutrition, varieties, cooking effect on nutrients, contaminants, and health relationships
Weaver & Marr. White Vegetables: A forgotten source of nutrients. Purdue roundtable executive summary. *Adv. Nutr*. 2013; 4:318S–326S.	Nine reviews of nutrients provided by white vegetables, with some specific to potatoes, including mineral nutrition, fiber, and glycemic response and diabetes
Yoshimatsu, et al. [Current status in the commercialization and application of genetically modified plants and their effects on human and livestock health and photoremediation]. *Yakugaku Zasshi*. 2012; 132 (5):629–274.	Summary of developments with genetically modified plants, such as use for nutraceuticals, medical curatives, and for production of oral vaccines
Zaheer & Akhtar. Recent advances in potato production, usage, nutrition – a review. *Crit Rev Food Sci Nutr*. June 2012.	General summary of potato nutrition, generation and mitigation of acrylamide, and other risks from potato consumption (e.g., allergies, alkaloids). Some health benefit‐related studies referenced
Systematic Reviews	
Al‐Khudairy, et al. Dietary factors and type 2 diabetes in the Middle East: what is the evidence for an association? *Nutrients*. 2013; Sep 26; 5(10): 3871–3897.	Identified 17 studies, mainly observational, examining association between dietary factors with T2DM. Only 2 studies (case–control and cross‐sectional) reported a significant positive association between potatoes and T2DM risk
Vlachojannis, et al. Medicinal uses of potato‐derived products: a systematic review. *Phytother Res*. 2010; 24 (2): 159–162.	Identified 5 clinical trials on medical use of potato extracts (e.g., oralfor dyspepsia, topical for perianal dermatitis). Only one RCT, which suggested an oral potato proteinase inhibitor may reduce food intake at subsequent meals
Yusof, et al. Dietary patterns and risk of colorectal cancer: A systematic review of cohort studies (2000–2011). *Asian Pac J Cancer Prev*. 2012; 13(9): 4713–4717.	Concluded a Western dietary pattern was associated with increased risk of colorectal cancer, whereas a prudent diet was not. Although Western diet was referenced as meat and potatoes, no specific data on potato intake was provided in most studies
Meta‐Analyses	
Mozaffarian, et al. Changes in diet and lifestyle and long‐term weight gain in women and men. *N Engl J Med*. 2011; 364 (25):2392–2404.	Dietary factors with the largest positive association with 4‐year weight gain in three large US cohorts included potato chips and potatoes
Nettleton, et al. Meta‐analysis investigating associations between healthy diet and fasting glucose and insulin levels and modification by loci associated with glucose homeostasis in data from 15 cohorts. *Am J Epidemiol*. 2013; 177 (2):103–115.	Utilized data from 15 US and European cohort studies to construct a diet score, with fried potatoes a component of an unfavorable score. Excluded baked, boiled, or mashed white potatoes in the assessment, although the cohort data included these foods

**Figure 1:**
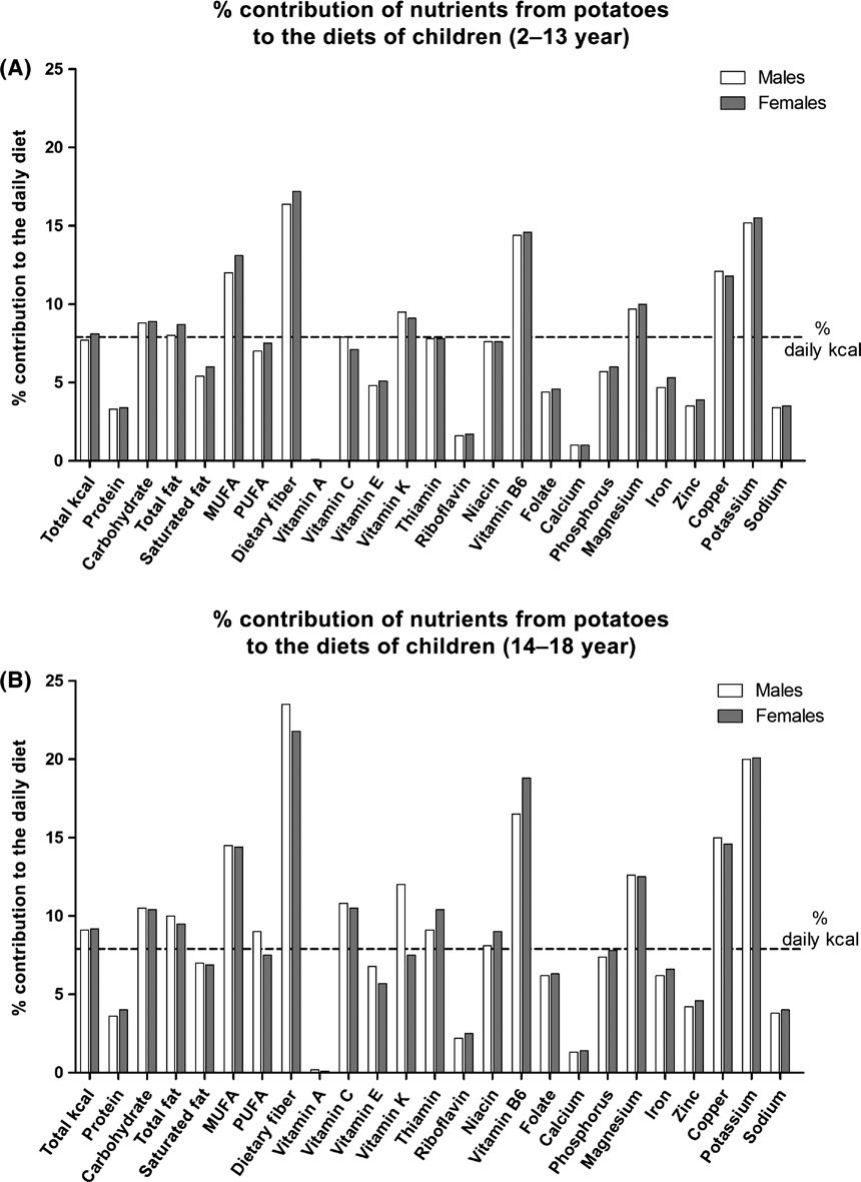
Percent contribution of nutrients from white potatoes, French fries, and oven‐baked fries compared to total calories for children age 2–13 years (A) and 14–18 years (B) using National Health and Nutrition Examination Survey (NHANES) 2003–2006 (Freedman and Keast 2011). Intakes for males and females are shown in solid white and gray bars, respectively. Percent total calories provided by potato foods is shown in the first column and represented by the horizontal dotted line.

Freedman and Keast (2011) assessed the contribution of white potatoes, French fries, and oven‐baked potatoes in the diets of children. Potatoes accounted for between 7.7% and 9.2% of daily energy intakes in children, and provided a higher average contribution of dietary fiber, vitamin B6, magnesium, copper, potassium, and MUFA than calories, and less sodium and saturated fats (Fig.**** 1A and B). In particular, potatoes delivered between 16.4% and 23.5% daily intakes of dietary fiber and between 15.2% and 20.1% of potassium to children's diets, two nutrients of concern in the U.S. population. Similar to the findings in children, studies with adults using National Health and Nutrition Examination Survey (NHANES) 2003–2006 (Freedman and Keast 2012) and 2009–2010 (Storey and Anderson 2013) have shown that potatoes and potato products provided more fiber, vitamin B6, and potassium than the corresponding energy contribution. Potatoes also provided substantial vitamin C, MUFA, and PUFA.

King and Slavin (2013) reviewed the role of the white potato in meeting dietary recommendations and noted that only ~1/3 of the total fat in white potatoes is in the form of saturated fatty acids (King and Slavin 2013). Moreover, potatoes have less lipid than that found in rice (0.2%) and pasta (0.9%), and have a higher biological value of protein than soybeans or legumes. Drewnowski 2013; reviewed the nutrient contribution of potatoes with respect to cost using the Affordable Nutrition Index, and report that, of the foods evaluated only white potatoes (boiled and baked) and carrots provide the three key characteristics of nutrient density, affordability, and consumer acceptance (Drewnowski 2013).

Potatoes are generally considered a high glycemic index (GI) food; however, in a recent review it was reported that the actual GI for potatoes commonly consumed in North America ranges from 56 to 94, indicating that not all potato preparations have a high GI (Anderson et al. 2013). The highest GI values are noted for mashed potatoes, although reported values vary considerably, with baked potatoes, French fries, and potato crisps generally having GI values in the medium range. The role of GI in health outcomes is controversial: recent assessments indicate there is only a moderate body of inconsistent evidence to support the relationship between high GI and type 2 diabetes; and that the amount of evidence regarding a link between GI and cardiovascular disease has not been conclusive (U.S. Department of Agriculture and U.S. Department of Health and Human Services, 2010).

Epidemiological evidence has shown an association between higher intakes of potato products and weight gain (Mozaffarian et al. 2011), but these studies have not separated the effect of potato consumption from the effect of components added during cooking/processing, such as fat, or other foods consumed with the potato. In our literature assessment, only one RCT that attempted to evaluate the specific role of potatoes as part of a weight‐loss diet was identified (Randolph et al. 2014). In that study, 90 overweight men and women were randomly assigned to one of three 12‐week interventions: (1) low GI energy reduced (−500 kcal/day) diet; (2) high GI energy reduced (−500 kcal/day) diet; or (3) habitual diet (control). All subjects were counseled to consume 5–7 servings of potatoes per week from a provided allotment of 6 russet and 3 red potato varieties weekly. All groups lost a modest, but statistically significant, amount of weight (average of −1.5 to 2.3 kg). As a result of these findings, the authors suggest potatoes do not interfere with weight loss or cause weight gain as part of a usual diet.

Several studies have investigated the effects of potato chips on markers of appetite/satiety, which are often linked to weight management. Potato chips were compared to other snack options on short‐term appetite and energy intake in 26 normal‐weight children, 8–11 years, and reported to lead to increased cumulative food intake compared with raisins or grapes, but lower than cookies (Patel et al. 2013). In another study, children who ate a snack of cheese and vegetables consumed ~72% fewer calories compared to children provided with potato chips in a short‐term assessment (Wansink et al. 2013). Tey et al. (2012) demonstrated that habitual consumption of energy‐dense snack foods (hazelnuts, chocolate milk, or salted potato chips) resulted in a decrease in sensory‐specific satiety over a 12‐week consumption period (Tey et al. 2012). These effects were not specific to potato chips alone, and results were compared to a control group that did not receive snack foods. Therefore, the effect of potato chip consumption as a snack on long‐term energy intake has not been thoroughly explored.

### Potato MRPs and health outcomes

The systematic literature search on MRPs from potato(es) and health outcomes yielded few studies. Vesper et al. (2005) conducted a pilot validation study in six generally healthy, nonsmoking adults to determine if acrylamide exposure from consumption of 3 ounces of potato chips daily over the course of a week increased the concentration of proposed biomarkers of acrylamide intake (Hb‐acrylamide and Hb‐glycidamide adducts) (Vesper et al. 2005). Adduct levels were detected in all subjects with increases of up to 46% for Hb‐acrylamide and 79% for glycidamide. The authors noted that the concentrations were within the range of background levels observed in previous studies (not specific to potato chip consumption). No results on health outcomes were reported.

In the only RCT identified that specifically examined dietary acrylamide exposure from potato, Naruszewicz et al. (2009) found that daily consumption of 160 g of potato chips (157 mg acrylamide) over four weeks induced a proinflammatory metabolic profile (increased blood concentrations of oxidized LDL‐C, interleukin‐6, and high sensitive C‐reactive protein) in generally healthy adults (Naruszewicz et al. 2009). Changes in these inflammatory biomarkers are potentially important because increased levels have been associated with risk for developing atherosclerosis. These results should be interpreted with caution, as the acrylamide intake level in this study was approximately three times higher than estimated intakes from a Western diet (~50 *μ*g/day) (Hagmar et al. 2005).

One observation study included in the evidence map was an analysis of a series of hospital‐based case–control studies in Italy and Switzerland (Bosetti et al. 2002). These studies were designed to describe the relationship between dietary intake of fried and baked potatoes and risk of developing various types of cancer (oral cavity/pharynx, esophagus, larynx, colon, rectum, breast, and ovary). The main findings from this study suggested a lack of an association between consumption of baked/fried potatoes and risk of cancer at several anatomical sites. This study was conducted in southern European populations that use different cooking processes than most population groups in the United States, which should be considered in translating the findings.

Three additional clinical trials of potatoes, MRPs, and health outcomes were identified in this literature review, but were not adequately designed to examine the effect of MRPs specifically from potatoes on health outcomes (Seiquer et al. 2006; Negrean et al. 2007; Abramsson‐Zetterberg et al. 2008). Two of these studies included potatoes in both dietary intervention groups using different methods of cooking (Seiquer et al. 2006; Negrean et al. 2007), and one included potatoes (French fries and potato chips) as part of a high‐fat diet (Abramsson‐Zetterberg et al. 2008). Further research is needed to characterize the MRP profile of potato and potato products. Additional research on the effect of different potato cooking methods on health outcomes is also needed, including prospective study designs that control for potential confounding of high‐protein foods (e.g., protein‐based melanoidins vs. starch‐based melanoidins).

### Benefits‐risk of potatoes and MRPs

The traditional approach to risk assessment has been described as a scientific endeavor that follows a defined pathway: (1) hazard identification, (2) hazard characterization, (3) exposure assessment, and (4) risk characterization (European Food Safety Authority (EFSA), 2006; Tijhuis et al. 2012). The approach is primarily based on studies in animals with defined components used in subchronic or chronic dose–response studies. Historically, the risk from specific foods and food components has been assessed separately from benefits, and has been the domain of the toxicology experts. More recently, scientists and public health officials have recognized that the health‐related benefits from foods need to be considered as well as part of a risk assessment (Tijhuis et al. 2012).

The most notable summary of benefit‐risk is that of Seal et al. (2008), who published three case studies on acrylamide showing an approach to including benefit along with a risk assessment (Seal et al. 2008). These authors used potatoes as one of the case studies, summarizing its benefits as provision of energy, vitamins C and B6, magnesium, and fiber (particularly when skins remain intact). However, the authors did not address potassium, a nutrient of need in the U.S. diet, or importance of potatoes in fiber nutriture even when skins are not attached. Increased potassium intake is thought to be the largest explanation for the decrease in blood pressure that occurs with consumption of the Dietary Approaches to Stop Hypertension (DASH) dietary pattern, and it has been estimated that an average increase of 1.6 g/day of potassium could lead to a 21% decreased risk of stroke in the U.S. population, independent of blood pressure benefits (D'Elia et al. 2014). Furthermore, in adults, dietary fiber has been shown to be associated with decreased risk for chronic diseases (e.g., obesity and diabetes) (Liu et al. 2015). Clearly, assessment of the impact of decreased consumption of potatoes on potassium and fiber, and potential increases in health issues, is warranted in order to balance the assessment of risk from acrylamide. Seal et al. (2008) also commented on the relatively high GI of potatoes and potato products when eaten hot, and suggested a relationship between high GI foods and adverse health outcomes, but as described previously in this document, the role of GI and health outcomes is controversial and not strongly supported by current science.

The review by Seal et al. (2008) did note a specific benefit of commercial production of potatoes as compared to home cooking from their literature assessment, indicating that: “*In general terms, it can be stated that industrial cooking leads to a much better control of desired and undesired changes than home cooking*.” Studies analyzing for markers of MRP formation in home cooked foods have shown wide ranges in the types of MRPs. It is interesting to speculate that MRPs may be less variable in processed foods due to consistent manufacturing practices. This point is particularly important in relation to mitigation efforts on acrylamide, which have resulted in substantial decreases of this MRP in foods that are manufactured.

Although not directly included in benefit‐risk assessments, which generally define benefit in terms of a nutrition or health outcome that can be defined and quantified, another important ‘benefit’ associated with food is the sensory attributes that impact consumer acceptability. For example, fried and baked potatoes are well accepted by consumers from a sensory perspective (Tijhuis et al. 2012), thus supporting the consumption of the nutrients, potassium and fiber.

## Conclusions

To our knowledge, this is the first attempt to characterize the breadth and scope of human clinical research on potato MRPs and health outcomes. The available studies have primarily focused on the formation of acrylamide and not other MRPs. There appears to be a major gap in the current understanding of the impact of MRPs, and specifically MRPs from potatoes, on human health. Indeed, much of the information on potential human health effects of MRPs, both positive and negative, has been extrapolated from in vitro and animal studies. For example, Muscat and colleagues, in a series of in vitro experiments, have shown that MRPs are cytotoxic (i.e., toxic to living cells) (Muscat et al. 2009) and can generate reactive oxygen species (e.g., H_2_O_2_) that may be important in immune and inflammatory reactions (Muscat et al. 2007), although these findings have not been confirmed in human clinical studies.

In our assessment, we found the majority of studies on potatoes conducted to date have either been confounded by inclusion of other nonpotato foods, or have focused on specific forms of potatoes (e.g., potato chips). For example, only one study has been conducted associating daily consumption of a large quantity of potato chips with risk of developing atherosclerosis, but this study does not definitively prove a link between potato consumption and cardiovascular disease. In addition, some studies have used colored potatoes (e.g., purple), which contain phytochemicals that may confer health benefits not typically associated white/yellow potatoes; however, these varieties are not commonly consumed in the average Western diet. Many of the limitations of observational evidence should be taken into consideration as well when interpreting the data for public health messages around MRPs, such as acrylamide, and potato intake. Furthermore, the focus on a specific individual compound may not be the optimal approach to evaluating a class of compounds (e.g., dietary MRPs), which may have differing health outcomes in the context of food (e.g., potato) that provides nutritional benefit.

Maillard reaction products gained attention in 2002 with the identification of acrylamide occurring in foods high in reducing sugars and asparagine after heating (e.g., via the Maillard Reaction). Because acrylamide has been classified as a ‘probable carcinogen’ and a causative agent in damage to the nervous system when administered at very high levels, a worldwide effort was initiated to decrease acrylamide in foods [reviewed in (Pruser and Flynn 2011)]. Several recent in‐depth reviews on the occurrence, mitigation efforts, and risk from acrylamide have been published (HEATOX 2007; Lineback et al. 2012; Arvanitoyannis and Dionisopoulou 2014). While associations between acrylamide at high intake levels and chronic health disease risks (primarily cancer) have been shown, few, if any, human studies using acrylamide intakes representative of current consumption patterns have been conducted.

Potatoes and potato products play a significant role in providing nutrients. Specifically, fiber and potassium are nutrients of concern in the U.S. population, with nearly all Americans, both adults and children falling short of meeting their daily recommendations for intakes (Hornick et al. 2011; King et al. 2012; Weaver and Marr 2013). Consideration of the impact of decreased potato consumption on intake of nutrients, particularly fiber and potassium and the potential subsequent health effects, should be included in a benefit‐risk assessment. Given the predominance of potatoes in providing the nutrients to the diets of U.S. adults and children, further research is warranted before conclusions on benefit‐risk of potato consumption can be made.

1. Understanding the current acrylamide intake from potatoes given the substantial mitigation efforts.
2. Include assessments of decreased potassium and fiber consumption when modeling changes in potato consumption.
3. Better identify the MRPs in potatoes, specifically the melanoidins, and understand whether in vitro effects as antioxidants and modulators of microbiota translate to health outcomes in humans.

## Conflict of Interest

Biofortis Research, Addison, IL received financial support for the evidence mapping project from the Frozen Potato Products Institute. The views expressed in this article are those of the authors and were developed independent of the funding agency.

## Supporting information


**Appendix S1.** Evidence map search terms and additional summary tables.
