# Supplementary material for: Maillard reaction products and potatoes: have the benefits been clearly assessed?
Source: Food Sci Nutr. 2015 Sep 17;4(2):234–49. doi: 10.1002/fsn3.283 (PMC4779479; doi:10.1002/fsn3.283)
Supplement: Supplementary file 1 — Appendix S1. Evidence map search terms and additional summary tables. [file FSN3-4-234-s001.docx]

Supplemental Material

**Maillard Reaction Products (MRPs) and Potatoes: The need for a benefit-risk approach**

DeAnn J. Liska^1^, Chad M. Cook^1^, Ding Ding Wang^2^, John Szpylka^3^

^1^ Biofortis Innovation Services, Addison, IL

^2^ Consultant, New York, NY

^3^ Silliker, Chicago, IL

### Methodology

Literature searches were conducted in Medline® (January 1994 to April 2014) and Scopus® databases. All studies published in English language with human subjects were screened to identify articles relevant to the inclusion/exclusion criteria. The search strategy employed the National Library of Medicine’s (NLM) Medical Subject Headings (MeSH) keyword nomenclature developed for Medline®. Reference lists of selected reviews were also screened for additional publications. Unpublished studies, clinical trial databases and articles in the grey literature were not searched; therefore the strategy did not include assessment for unpublished data. References of key article were also mined for additional references.

Two searches were performed and combined for the MRP Evidence Map:

- Search 1: MRPs included search terms:
  - (Maillard or advanced glyc$ or glycation or thermal).mp. [mp=title, abstract, original title, name of substance word, subject heading word, keyword heading word, protocol supplementary concept word, rare disease supplementary concept word, unique identifier]
  - Acrylamide.mp. or Acrylamide
- Search 2: Diet and Health Outcomes search
- (diet$ or food).mp. [mp=title, abstract, original title, name of substance word, subject heading word, keyword heading word, protocol supplementary concept word, rare disease supplementary concept word, unique identifier]

The potato and MRP Evidence Map included the terms above as well as:

- *Potato or Potatoe or Potatoes).mp. [mp=title, abstract, original title, name of substance word, subject heading word, keyword heading word, protocol supplementary concept word, rare disease supplementary concept word, unique identifier*
- *Solanum tuberosum*

### Study Selection and Eligibility Criteria

All abstracts identified through the literature search were screened based on eligibility criteria with a low threshold to exclude irrelevant abstracts, such as animal or *in-vitro* studies, as well as studies that did not investigate a health relationship (health relationship included direct health outcome or physiological markers relevant to human health outcomes. A web-based citation screening tool, Abstrakr^TM^ (http://abstrackr.cebm.brown.edu/), was used to facilitate the abstract screening process. Full-text articles were retrieved for studies that could not be resolved based on the title, keywords, and the abstract review process.

- **Inclusion Criteria:**
  - Ovid MEDLINE® (<Jan. 1994 to April 2014) and Scopus Databases
  - Randomized controlled clinical trial, cohort, case report, or cross-sectional studies
  - Studies that assessed a health aspect/ relationship of MRPs
  - Studies that included dietary exposure or food with MRPs
- **Exclusion Criteria:**
  - Studies published in a language other than English
  - Studies with no health relationship in humans
  - *In-vitro* studies or studies in animals
  - No documentation of dietary exposure of interest (e.g., MRP)
  - Studies in infants or pregnant women
  - Addresses, bibliographies, interviews, lectures, comments, dictionary entries, editorials, or guidelines
  - Reviews, meta-analyses, systematic reviews

The individual searches resulted in >50,000 titles each. The combined search resulted in 1896 titles, and after review of abstracts against the inclusion/exclusion criteria, 292 potential studies were identified. A subsequent full-text review of these studies against the inclusion/exclusion criteria resulted in a 41 studies were included in the final evidence map: 18 clinical trials, 23 observational studies. The majority of excluded studies were on acrylamide exposures in populations or food chemistry papers on techniques to determine or reduce acrylamide during food processing. Selected recent reviews that represented the literature on acrylamide were identified and included in the findings discussion. For the potato and MRP evidence map, the MRP initial search results were crossed with the search using terms for potato. The clinical trials are shown in Table A Representative publications Identifying the Prospective and Case-Control Cohorts assessed for MRPs and health outcomes are provided in Tables B and C. Also see recent reviews below for human observational studies on acrylamide.

Hogervorst JG, Baars BJ, Schouten LJ, Konings EJ, Goldbohm RA, van den Brandt PA. The carcinogenicity of dietary acrylamide intake: a comparative discussion of epidemiological and experimental animal research. *Crit Rev Toxicol.* 2010 Jul;40(6):485-512.

Pelucchi C, La Vecchia C, Bosetti C, Boyle P, Boffetta P. Exposure to acrylamide and human cancer--a review and meta-analysis of epidemiologic studies. *Ann Oncol*. 2011 Jul;22(7):1487-99.

Tessier FJ, Birlouez-Aragon I. Health effects of dietary Maillard reaction products: the results of ICARE and other studies. *Amino Acids.* 2012;42:1119-1131.

**PRISMA Chart on the search and literature assessment for MRPs and human health outcomes (above) and potatoes and MRPs (below).**

**Table A. Randomized Controlled Clinical Trials (RCT) on MRPs and Health Outcomes published from January 1994 to April 2014.**

| **Citation** | **Study Design** | **Population,**  **Age (y), &**  **COUNTRY** | **Test**  **Food / Diet** | **Control**  **Food/ Diet** | **Duration** | **Outcomes** | **Comments** |
| --- | --- | --- | --- | --- | --- | --- | --- |
| Birlouez-Aragon I, et al*. AJCN*. 2010; 91:1220-6 | RCT  X-over | 62 healthy adults (50% male)  Age 18-24 (mean, 19)  FRANCE | Standard diet (STD)  Rich in MRP (coffee, grilled/ roasted foods, corn flakes, dry cookies, well-baked bread) | Steamed diet (STM)  Low in MRP  (raw foods, team, mildly baked bread, sponge cake, steamed corn flakes) | 1 wk/diet  10-d washout | 10% higher caloric intakes, carbohydrates & fats, lower vit. C intakes during STD diet; Mean CML in STD diet: 5.4mg/d; STM diet: 2.2 mg/d  STM resulted in significantly lower tChol and HDL-C, fasting insulin, HOMA; and higher vit C & E, and ubiquinone. No difference in LDL-C, fasting glucose, Hb1c, antioxidant assays. Lower plasma omega-3 fatty acids on STD diet. | Negative effect of higher MRP diet  Confounded due to lack of control for caloric intake and fatty acids suggest very different diet compositions.  Changes could be due to calories alone or types of foods, and not MRP specific. |
| Correa TAF, et al.  *Plant Foods Hum Nutr.* 2012;  67:277-82. | RCT  X-over | 20 healthy adults  Habitual coffee drinkers  (30% male)  Age 49±9  BRAZIL | Medium roast coffee (MR), paper-filtered  482±61 mL/d  334mg CGA/150mL  Higher MRP | Medium light roast coffee (MLR), paper-filtered  482±61 mL/d  210mg CGA/150mL  Lower MRP | 4 wk/diet  No washout  1 wk run-in | Outcomes: tAntiox, ORAC, oxLDL, PGF2a, SOD, GPx, Catalase  Both coffees showed significant increases in antioxidant activities and did not change lipid peroxidation markers compared to baseline. No significant difference between coffee diets. | No difference between high MRP and lower MRP coffees |
| Seiquer I, et al.  *Am J Clin Nutr.* 2006;  83:1082-88. | RCT  X-over | 20 healthy children recruited;  18 completed  (100% male)  Age 12.4±0.34  SPAIN | Brown diet (BD), rich in MRP containing foods (fried & breaded foods, corn flakes, chocolates, baked products) | White diet (WD) free as much as possible of MRPs  Similar energy, foods, nutrients, as test diet | 2 wk/diet  40-d washout | BD showed 47% higher fecal excretion of nitrogen, 12% lower apparent nitrogen absorption, 6% lower nitrogen digestibility. Apparent nitrogen retention and utilization of ingested nitrogen and serum biochemical variables related to nitrogen did not differ significantly | Negative impact of higher MRP on in vitro protein digestibility. No difference on utilization.  Potatoes on both diets (boiled/ baked vs. fried), however differences in the food forms could have non-MRP specific effects |
| Garcia MM, et al  *Mol Nutr Food Res*.2009; 53:1551-60.  ***Duplicate with Seiquer, 2006*** | See Above | See Above | See Above | See Above | See Above | ~1.4-fold increase in iron fecal excretion and ~2.7-fold decrease in bioavailability with BD diet compared to WD diet.  Serum biochemical markers related to iron metabolism were not different. | Negative impact on dietary iron bioavailability.  See above for caveats |
| Delgado-Andrade C. et al. *Nutrition.* 2010; 27:86-91.  ***Duplicate with Seiquer, 2006*** | See Above | See Above | See Above | See Above | See Above | No differences in wt & ht, nutrient intakes (e.g., phosphorus), except fat, vit E. Significantly higher fecal phosphorus excretion after BD diet (decreased mineral absorption). Serum Ca, phosphorus PTH and AP not different | Negative impact on dietary phosphorus absorption.  See above for caveats |
| Mesias M, et al.  *J Agric Food Chem*. 2009;  57:9532-38.  ***Duplicate with Seiquer, 2006*** | See Above | See Above | See Above | See Above | See Above | No difference in serum & urine biochemical measures of Ca status & bone metabolism  Deoxypyridinoline significantly lower after BD, suggesting less bone turnover | Mixed results  Null for most bone health markers except one, which was negative for BD  See above for caveats |
| Delgado-Andrade C, et al*. Amino Acids.* 2011; 43:595-602.  ***Duplicate with Seiquer, 2006*** | See Above | See Above | See Above | See Above | See Above | BD diet resulted in higher CML input (11.28mg/d) vs. WD (5.36 mg/d). Fecal excretion of CML was also greater with BD diet (3.52 mg/d) vs. WD (1.23 mg/d) and proportional to dietary intakes. Urinary CML not different. | NA  See above for caveats |
| Harcourt BE, et al*. Kidney Intl.* 2011;  80:190-98. | RCT  X-over | 11 overweight & obese adults  100% male  Age 30±9  AUSTRALIA (?) | High AGE meal  Food prepared by frying, toasting, heating | Low AGE meal  Matched in kcal and foods, preparations changed to steaming, poaching, fresh/raw | 2 wk/diet  4 wk washout | Renal function and inflammatory profile were improved after the Low AGE diet vs. High AGE diet | Negative effect of higher MRP  Disposition of subjects not provided, and diets well matched. |
| Negrean M, et al.  *Am J Clin Nutr.* 2007;  85:1236-43. | RCT  Single-blind  X-over | 20 T2DM inpatients  70% males  Age 55.4±2.2  GERMANY | High AGE meal  200g chicken  150g potatoes  100g carrots  200g tomatoes  15g vegetable oil  580 kcal total  Food was fried or broiled | Low AGE meal  Same meal as High AGE, with food prepared by steaming or boiling | 6-d on standardized diabetes diet  Test diets on day 4 and 6 | High AGE vs. Low AGE meal decreased FMD by 36.2% vs. 20.9%, respectively (P<0.001). High AGE meal showed impairment of microvascular function and increased serum AGE and markers for endothelial dysfunction and oxidative stress. | Negative effect of higher MRP  Potatoes included in both diets. Highly compromised population with complications of diabetes. |
| Stirban A, et al. *Ann NY Acad Sci.* 2008; 1126:276-79.  **Possible Duplicate with Negrean, 2007** | RCT  Single-blind  X-over | 20 inpatient adults with 8.7±1.7 y T2DM duration;  Age 55.9±2.0  GERMANY | See above | See above | See above | See above | See above |
| Stirban A, et al.  *Diabetes Care.* 2012; 36:1278-82. | RCT  Double-blind  X-over | 19 T2DM patients  Age 54 | AGE beverage  Beta-lactoglobulin and dextrose heated beverage  120,000 U CML/serve | Control beverage  Prepared the same but without dextrose added. 19,400 U CML/serve | Single dose  7-d washout | AGE-beverage decreased FMD by 80% compared to 27% for control. Significant decrease in nitrite and nitrate, significant increase in CML following AGE beverage. No change in microvascular function by either beverage. | Negative effect of higher MRP  Single dose in patients with frank diabetes. |
| Uribarri J, et al.  *J Am Soc Nephrol*. 2003; 14:728-31. | RCT  Parallel | 26 nondiabetic, stable, renal failure patients recruited;  18 completed  33% males  Age NR  USA | High AGE diet  Patients instructed to roast, broil, and oven fry foods | Low AGE diet  Patients instructed to boil, poach, steam, stew foods and avoid fried foods | 4 wk | Low AGE diet decreased serum CML, MG, CML-LDL, CML-apoB, and dialysate CML and MG outputs.  High AGE increased serum CML, MG, CML-LDL, CML-apoB, and dialysate CML output | Negative effect of higher MRP  Relatively short report with limited details on subjects provided. |
| Abramsson-Zetterberg L, et al.  *Mutation Res.* 2008;  653:50-56. | RCT  Parallel | 24 healthy adults  42% males  Age 46 (24-60)  SWEDEN | High-heated food group (HighHF)  Subjects counseled to choose fried foods; supplied with French fries, potato crisps, biscuits, and crisp bread. | Low heated food group (LowHF)  Subjects counseled to minimize heated foods; supplied with fresh potatoes, buns, and white bread. | 4 d dietary intakes | HighHF diet resulted in increased frequency of micronucleated young erythrocytes, which decreased from baseline after the LowHF diet. Mean total AA intake estimated 3000±450 mcg/person for HighHF diet, and 20±10 mcg/person for LowHF diet. | Negative effect of higher MRP  Differences can also be attributed to other differences in the diets, such as fat, which was higher in the High HF diet. |
| Vikström AC, et al. *Toxicol Sci.* 2010;  119(1):41-49.  **2 studies; 1 duplicate with Abramsson-Zetterberg, 2008.** | RCT  Study1: Parallel  Study 2:Single arm | 19 non smokers  37% males  Age 46 (24-60)  Study 2: duplicate of 9 subjects (non-smokers)  SWEDEN | Study 1: HighAA diet; Food heated at high temperatures  760 mcg/day for 4 days; Medium AA diet 160 mcg/day for 28 days  Study 2: 500 g potato chips for 28 d after 2 wk run-in | Study 1: LowAA diet; Food heated at medium to low temperatures  Study 2: Run-in, 500 g boiled potatoes for 2 wk | Study 1: 4-d  Study 2: 2 wk run-in, 20-d test diet | Significant increase of blood levels for AA adducts and genotoxic metabolite glycidamide after High AA and medium (28-d potato chip) AA diets. | Negative effect of higher MRP  Study subjects not well characterize. Intakes of AA reasonable to average diets. |
| Vlassara H, et al.  *PNAS.* 2002;  99:15596-601. | RCT  Study 1 X-over  Study 2  Parallel | Non-smoking T1 diabetic and T2DM adults  Study 1: 11  Age: 52±2  Study 2: 13  Age ~62  USA | High AGE diet  Diet followed NCEP Step 1 & AHA plans with high cooking times, AGE content 16.3±3.7x10^6^  U/d  Diets matched to subject for wt. maintenance | Low AGE diet  Comparable diet to High AGE with lower cooking times; AGE content 3.7±1.2x10^6^  U/d  Diets matched to subject for wt. maintenance | Study 1:  2 wk/ diet  1-2 wk washout  Study 2: 6 wk | Study 1: Serum AGE increased 64.5% with High-AGE diet, decreased 30% on Low-AGE diet; TNF-α/β-actin mRNA ration higher on High-AGE vs. Low-AGE. No changes in blood glucose or lipid values.  Study 2: Significant increase in TNFα (86.3%), CRP (35%), VCAM-1 (4%), and AGE-LDL-C (32%), with declines of each on Low-AGE diet (20%, 20%, 20%, 33%, respectively). Serum AGE increased by 28.2% on High-AGE diet, and decreased 40% on Low-AGE diet. | Negative effect of higher MRP  Meals appeared to vary in food type, and thus likely varied in fat, protein, etc. Study has advantage of diets designed for wt maintenance. |
| Naruszewicz M, et al. *Am J Clin Nutr.* 2009; 89:733-7. | CT  Single arm | 14 healthy adults  43% males  Age 35 (22-56)  SWEDEN or POLAND | 160g potato chips (878 kcal, 1374 mg sodium, 980, 157mcg AA, <1% TFA) | 400g boiled potatoes with fat and salt at amounts found in potato chips and avoid potato chips | 2 wk run-in on boiled potatoes; 28-d potato chips; 28-d boiled potatoes | Significant increase in blood AA-Hb adducts, oxLDL, hs-IL-6, hsCRP, and reactive oxygen radical production by monocytes, lymphocytes, and granulocytes, and an increase in CD14 in macrophages after potato chips. No change in lipids or glucose. | Negative effect of higher MRP  Diets not well described. Single arm with shorter run-in as control/baseline, not randomized. Data did not return to baseline for all parameters during 3^rd^ phase (control). |
| Uribarri J, et al. *Diabetes Care*.  2011; 34:1610-16. | RCT | 18 T2DM adults with usual diet high in AGE (>20 AGE Eq/d)  Age 61±4;  18 healthy adults  Age 67±1.4  USA | 12 T2DM adults on AGE-restricted diets- AGE restricted diet (boil, poach, stew, or steam food, limiting AGE intake by ~40-50%);  6 T2DM adults on usual diet (>20 AGE Eq/d) | isocaloric AGE-restricted diet (boil, poach, stew, or steam food, limiting AGE intake by ~40-50%) | 4 mo | Insulin and HOMA, leptin and other serum inflammation and antioxidant markers decreased with AGE restricted diets. | Negative effect of higher MRP  Diets not well described or monitored. |
| Vesper HW, et al. In: Chem Safety Food Acryl. Ed. Friedman, Mottram. Springer Sci & Bus Media Inc.  2010; pg. 89-95. | CT  Single-arm | 6 healthy non-smoking adults;  Age 18 or older  USA | 3 ounces of potato chips (84g; 1,373 mcg/kg AA) for 115 mcg AA/person/d | none | 7 d | Increase of serum AA adducts and glycidamide adducts increased by 46% and 79%, respectively.  None of the adducts were within the range seen in smokers, and the amount of consumption estimated at ~ 3x higher than average AA content of average potato chip consumption in the U.S. | Biomarker study only, no health outcome.  Not a peer-reviewed publication. CDC substudy of larger study. |

Abbreviations: AA, acrylamide; AGE, advanced glycosylated end-product; AGE Eq, 1000 kU AGE; AHA, American Heart Association; AP, alkaline phosphatase; ApoB, apo-lipoprotein-B; BD, brown diet; Ca, calcium; CDC, Center for Disease Control; CGA, chlorogenic acid; CML, N-ε-carboxymethyl-lysine; CRP, C-reactive protein; d, day; Eq, equivalents; FMD, flow-mediated dilation; g, gram; GPx, glutathione peroxidase; Hb, hemoglobin; HOMA, homeostasis model assessment; HDL-C, high density lipoprotein cholesterol; HighHF, high-heated food; hs-IL-6, high sensitivity interleukin 6; ht, height; kcal, kilocalorie; kU, kilo-units; LDL-C, low-density lipoprotein cholesterol; LowHF, low-heated food; mcg, microgram; mg, milligram; MG, methylglyoxal-derivatives; mo, month; MR, medium roast coffee; mRNA, messenger Ribonucleic acid; MLR, medium light roast coffee; MRP, Maillard reaction product; MRPs, Maillard reaction products; NCEP, National Cholesterol Education Program; NR, not reported; ORAC, oxygen radical absorption capacity; oxLDL, oxidized low density lipoprotein; PGF2a, prostaglandin F-2-alpha; PTH, parathyroid hormone; RCT, randomized clinical trial; STD, Standard Diet; SOD, superoxide dismutase; T1, Type 1; T2DM, type 2 diabetes mellitus; tAntiox, Total antioxidant capacity; tChol, Total cholesterol; TFA, *Trans* fatty acids; TNF-a, tumor necrosis factor alpha; U, units; U.S. United States; VCAM-1, vascular cell adhesion protein 1; vit, vitamin; WD, white diet; wk, week(s); wt, weight; X-over, crossover; y, years

**Prospective Cohort Studies on MRPs and Health Outcomes published between January 1994 and April 2014.**

| **Citation** | **Cohort Name** | **Duration (y)** | **Population** | **Exposure Assessment** | **MRP** | **Outcome** | |
| --- | --- | --- | --- | --- | --- | --- | --- |
| Burley VJ, et al. *Brit J Cancer*. 2010; 103:1749-54. | UK Women’s Cohort Study | 11 | 33,731 | FFQ | Mean AA intake 10 mcg/d, inter-quartile 10-21 mcg/d. Highest contributors: Potato chips, bakery goods, potato crisps, bread, biscuits and coffee. | No evidence of association between AA and postmenopausal breast cancer risk. Suggestion of weak positive association between dietary AA and premenopausal breast cancer risk. | |
| Hogervorst JG*, J Nutr.* 2008; 138:2229-36. | The Netherlands Cohort Study on diet and cancer | 13.3 | Case-Cohort analysis; Random subcohort of 5000 men & women aged 55-69 | FFQ | Mean AA intake 21.7±12.1 mcg/d. Foods used for analysis included: Potato chips, Dutch spiced cake, French fries salty snacks crisp bread, cookies, corn flakes, coffee | With every 10 mcg increment increase, HR for colorectal, gastric, pancreatic, and esophageal cancer risk were 1.0, 1.02, 1.06, and 0.96, respectively. No association with gastrointestinal cancers observed. |  |
| Hogervorst JG, et al. *Am J Clin Nutr*. 2008; 87:1428-38. | The Netherlands Cohort Study on diet and cancer | 13.3 | Case-Cohort analysis; Subcohort of 5000 men & women aged 55-69 | FFQ | Highest quintile mean AA 40.8 mcg/d); lowest 9.5 mcg/d. Foods used for analysis included: Potato chips, Dutch spiced cake, French fries salty snacks crisp bread, cookies, corn flakes, coffee | Highest quintile to lowest showed some indication of increased renal cell cancer risk (HR, 1.59). No risk seen with bladder (HR, 1.15) or prostate (HR, 1.06) cancers, although a nonsignificant positive trend reported for prostate cancer. |  |
| Hogervorst JG, et al*. Cancer Epidemiol Biomarkers Preven*. 2007; 16:2304-13. | The Netherlands Cohort Study on diet and cancer | 11.3 | Case-Cohort analysis; Random subcohort of 2589 women aged 55-69 | FFQ | Highest quintile mean AA 40.2 mcg/d, lowest 8.9 mcg/d. Foods used for analysis included: Potato chips, Dutch spiced cake, French fries salty snacks crisp bread, cookies, corn flakes, coffee | Highest quintile to lowest showed increased risk of postmenopausal endometrial (HR, 1.29) and ovarian (HR, 1.78) cancers. Strongest effect seen among never-smokers. No increased risk of breast cancer (HR, 0.93). |  |
| Mucci LA, et al. *Int J Cancer.* 2005; 118:169-173. | Swedish Mammography Cohort | ~15 | 61,467 women;  823072 person year | FFQ | Mean AA intake 24.6 mcg/d  Highest contributory foods: coffee, fried potato products, crisp bread, other breads. | No association between AA intakes and colorectal (RR, 0.9), colon (RR, 0.9), or rectal (RR, 1.0) cancers. |  |
| Larsson SC, et al. *Am J Epidemiol.* 2008; 169:376-81. | Swedish Mammography Cohort | 17.5 | 61,433 | FFQ | Mean AA intake 24.6 mcg/d. Highest contributory foods: coffee, whole-grain bread, crisp bread, breakfast cereals, cookies/buns, and fried potatoes. | No positive association with breast cancer risk. |  |
| Larsson SC, et al *Int J Cancer.* 2009; 124:1196-99. | Swedish Mammography Cohort | 17.7 | 61,226 | FFQ | Highest quartile AA 33.8 mcg/d, lowest 15.9 mcg/d. Highest contributory foods: coffee, whole-grain bread, crisp bread, breakfast cereals, cookies/buns, and fried potatoes | No positive association with endometrial cancer risk. |  |
| Larsson SC, et al. *Cancer Epidemiol Biomarker Prev*. 2009; 18:994-97. | Swedish Mammography Cohort | 17.5 | 61,057 | FFQ | Highest quartile AA 32.5 mcg/d, lowest 16.9 mcg/d. Highest contributory foods: coffee, crisp bread, white, rye and whole grain breads, pan-fried potatoes, potato chips, French fries, biscuits, cakes, crackers, chocolate, pancakes, cereals and meatballs. |  |  |
| Larsson SC, et al. *Eur J Cancer.* 2009; 45:513-16. | Cohort of Swedish Men | 9.3 | 45,306 | FFQ | Highest quartile AA ≥41.7 mcg/d, lowest 24.6 mcg/d. Highest contributory foods: coffee, crisp bread, white, rye and whole grain breads, pan-fried potatoes, potato chips, French fries, biscuits, cakes, crackers, chocolate, pancakes, cereals and meatballs. | No evidence of association between dietary AA and colon rectum cancer risk |  |
| Larsson SC, et al.  *Cancer Epidemiol Biomarker Prev*. 2009; 18(6):1939-41. | Cohort of Swedish Men | 9.1 | 45,306 | FFQ | Highest quintile AA 49.8 mcg/d, lowest 23.7 mcg/d. Highest contributory foods: coffee, crisp bread, white, rye and whole grain breads, pan-fried potatoes, potato chips, French fries, biscuits, cakes, crackers, chocolate, pancakes, cereals and meatballs. | No evidence of a positive prostate cancer risk with dietary AA |  |
| Mucci LA, et al. *JAMA.* 2005; 293:1326-27. | Swedish Women’s Lifestyle and Health Cohort | ~11  (490,000 person-years) | 43,404 | FFQ | Mean age at baseline 39 y, 9% postmenopausal women. Highest quintile AA 44 mcg/d, lowest 12 mcg/d. Highest contributory foods: coffee, crisp bread, white, rye and whole grain breads, pan-fried potatoes, potato chips, French fries, biscuits, cakes, crackers, chocolate, pancakes, cereals and meatballs. | No association between AA intake and breast cancer. |  |
| Schouten LJ, et al. 2009; *Am J Epidemiol.* 170(7):873-84. | The Netherlands Cohort Study | 16.3 | 120,852 | FFQ | Highest quintile mean AA 40.2 mcg/d, lowest 8.9 mcg/d. Foods used for analysis included: Potato chips, Dutch spiced cake, French fries salty snacks crisp bread, cookies, corn flakes, coffee | Dietary AA intake was not positively associated with risk of head-neck and thyroid cancer, except with oral cavity cancer risk for female nonsmokers, but the numbers were small. A negative association for males was indicated. |  |
| Wilson KM, et al. *Int J Cancer.* 2012; 131: 479-87. | Health Professionals Follow-up Study | 10 | 47,896 | FFQ | Highest quintile AA 40.1 mcg/d, lowest 10.5 mcg/d. Highest contributory foods: French fries, potato chips, breakfast cereal and coffee | No evidence that AA intake, within the U.S. diet, is associated with prostate cancer risk. |  |
| Wilson KM, et al. *Cancer Epidemiol Biomarker Prev*. 2010; 19:2503-15. | Nurse's Health Study II | 26 | 88,672 | FFQ | Highest quintile AA 40.1 mcg/d, lowest 10.5 mcg/d. Highest contributory foods: English muffins/rolls/bagels, breakfast cereal, coffee | No association between dietary AA intake and breast cancer |  |

Abbreviations: AA, acrylamide; d, day; FFQ, Food Frequency Questionnaire; HR, Hazard Ratio; mcg, microgram; mo, month; MRPs, Maillard reaction products; RR, relative risk; T2DM, type 2 diabetes mellitus; UK, United Kingdom; U.S., United States; y, years

**Table B. Control and Cross-sectional Studies on MRPs and Health Outcomes published between January 1994 and April 2014.**

| **Citation** | **Year** | **Study design** | **Cohort** | **Sample size** | **Outcome** |
| --- | --- | --- | --- | --- | --- |
| Chao PC, et al. *Eur J Nutr*. 2010;49:429-34. | 2010 | Case Control | Taiwan Cohorts | 74 healthy adults;  50 low AGE intake, and 68 high AGE intake adults with T2DM | Diabetics with high AGE intake (7-day diet diary) had significantly increased plasma AGE, HbA1_C_, LDL-C and glycated LDL. |
| Hogervorst JG, et al. *J Nat Cancer Inst*. 2009; 101:651-62. | 2009 | Nested Case Control | The Netherlands Cohort | 120,852 participants | After 13.3 y follow-up, no association with lung cancer risk in men, but inverse association in women, most strongly for adenocarcinoma. |
| Hogervorst JG, et al. *Cancer Epidemiol Biomarkers Prev.* 2009 May; 18(5):1663-6. | 2009 | Nested Case Control | The Netherlands Cohort Study | Subgroup of 5,000 subjects from the original group of 120,852 men and women aged 55-69 y | After 16.3 y follow-up, dietary AA intake was not associated with increased risk of brain cancer in the entire sub-cohort or in subgroups based on histology or smoking status. |
| Olesen PT, et al*. Int J Cancer* 2008;122(9):2094-100. | 2008 | Nested Case Control within Prospective Cohort | Danish Diet, Cancer and Health Study | 748 participants | Positive association between risk for ER+ breast cancer and AA-Hb adducts (acrylamide biomarker). |
| Pedersen GS, et al*. Breast Cancer Res Treatment*. 2009; 122:199-210. | 2009 | Nested Case Control | The Netherlands Cohort | 120,852 participants | Highest quintile of AA 36.8 mcg/d; lowest 9.5 mcg/d. No association for overall breast cancer or ER- breast cancer. A slight non-significant positive association between AA intake and ER+/PR+ breast cancer in never-smoking post-menopausal women was suggested. |
| Pelucchi C1, et al. *Int J Cancer*. 2003;105:558-560. | 2003 | Case Control | Italy and Switzerland | Oral cavity & pharynx:749 cases; 1,772 controls,  Esophagus: 395 cases; 1,066 controls,  Larynx:-527 cases; 1,297 controls,  Large bowel: 1,225 colon; 728 rectum; 4,154 control,  Breast: 2,569 cases; 2,588 controls,  Ovary: 1,031 cases; 2,411 controls | Assessed intake of fried/baked potatoes and cancers.  All the odds ratios (OR) for the highest vs. the lowest tertile of intake ranged between 0.8–1.1. No evidence of interaction with age, gender, alcohol and tobacco use was found. Data did not support an association between consumption of fried/baked potatoes and cancer risk. |
| Semba RD, et al. *Am J Hypertens*. 2008; 22:74-9. | 2008 | Cross sectional of Prospective Cohort | Baltimore Longitudinal Study of Aging (BLSA) | 493 participants | High AGE associated with an incr**e**ase in aortic pulse wave velocity reported for a subset of subjects. |
| Uribarri J, et al. *J Gerontol A Biol Sci Med Sci.* 2007; 62(4): 427-33 | 2007 | Cross-sectional | USA Cohort | 116 adults age 18-45 and 56 adults age 60-80 | Serum AGE indicators (CML and MG derivatives) were elevated in older participants and correlated with indicators of inflammation and oxidative stress across all ages. |
| Wirfält E, et al. Wirfält E, et al. *Eur J Clin Nutr.* 2008;62(3):314-23. | 2008 | Cross-sectional of larger Prospective Cohort | Malmö Diet and Cancer study | 70 non smoking  72 smoking  men and women | Positive association between Hb adducts and estimated AA intakes from foods observed in non-smoking men and smoking men and women, but not in non-smoking women. |
| Wilson KM, et al. *Int J Cancer*. 2009; 124;2384-2390. | 2001- 2002 | Population-based case-control study | Cancer of the Prostate in Sweden Study (CAPS) | 1499 cases  1118 controls | No significant associations between acrylamide exposure and risk of prostate cancer by stage, grade, or PSA level. Acrylamide Hb adducts and dietary intake of acrylamide (by FFQ) were moderately correlated, but not associated with risk of prostate cancer. |
| Xie J, et al. *Cancer Epidemiol Biomarkers Prev*. 2013; 22:653-60. | 2013 | Case Control | Nurses Health Study I & II | 263 participants | No evidence that AA exposure, as assessed by AA-Hb adducts, increases risk of ovarian cancer. |

**Abbreviations:** AA, acrylamide; AGE, advanced glycosylated end-product; CML, N-ε-carboxymethyl-lysine; CV, cardiovascular; d, day; ER+, estrogen receptor positive; Hb, hemoglobin; HbA1_C_, , hemoglobin A1c; HbA1_C_, LDL, low density lipoprotein; LDL-C, low density lipoprotein cholesterol;. mcg, microgram; MG, methylglyoxal-derivatives; mo, month; MRPs, Maillard reaction products; OR, odds ratio; PR+, progesterone receptor positive; T2DM, type 2 diabetes mellitus; USA, United States; y, years
